# Impact of ECG Findings and Process-Of-Care Characteristics on the Likelihood of Not Receiving Reperfusion Therapy in Patients with ST-Elevation Myocardial Infarction: Results of a Field Evaluation

**DOI:** 10.1371/journal.pone.0104874

**Published:** 2014-08-21

**Authors:** Kevin A. Brown, Laurie J. Lambert, James M. Brophy, James Nasmith, Stéphane Rinfret, Eli Segal, Simon Kouz, Dave Ross, Richard Harvey, Sébastien Maire, Lucy J. Boothroyd, Peter Bogaty

**Affiliations:** 1 Institut national d’excellence en santé et en services sociaux (INESSS), Montréal, Québec, Canada; 2 McGill University Health Center, Montréal, Québec, Canada; 3 St. Paul’s Hospital, Vancouver, British Columbia, Canada; 4 Institut universitaire de cardiologie et de pneumologie de Québec, Québec, Québec, Canada; 5 Sir Mortimer B. Davis Jewish General Hospital, Montréal, Québec, Canada; 6 Corporation d’Urgences-santé, Montréal, Québec, Canada; 7 Centre hospitalier régional de Lanaudière, Joliette, Québec, Canada; 8 Services préhospitaliers d’urgence en Montérégie, Longueuil, Québec, Canada; 9 Département de médecine préhospitalière, Hôpital Sacré-Cœur de Montréal, Montréal, Québec, Canada; 10 Université de Sherbrooke, Sherbrooke, Québec, Canada; 11 Centre hospitalier affilié universitaire Hôtel-Dieu de Lévis, Lévis, Québec, Canada; Scuola Superiore Sant’Anna, Italy

## Abstract

**Background:**

Many patients with ST-elevation myocardial infarction (STEMI) do not receive reperfusion therapy and are known to have poorer outcomes. We aimed to perform the first population-level, integrated analysis of clinical, ECG and hospital characteristics associated with non-receipt of reperfusion therapy in patients with STEMI.

**Methods and Results:**

This systematic evaluation of STEMI care in 82 hospitals in Quebec included all patients with a discharge diagnosis of myocardial infarction, presenting with characteristic symptoms and an ECG showing STEMI as attested by at least one of two study cardiologists or left bundle branch block (LBBB). Excluding LBBB, an ECG was considered a *definite* STEMI diagnosis if both cardiologists scored ‘certain STEMI’ and *ambiguous* if one scored ‘uncertain’ or ‘not STEMI’. Centers were classified according to accessibility to primary percutaneous coronary intervention (PPCI): 1) on-site PPCI; 2) routine transfer for PPCI; 3) varying mix of PPCI transfer and on-site fibrinolysis; and 4) routine on-site fibrinolysis. Of 3730 STEMI/LBBB patients, 812 (21.8%) did not receive reperfusion therapy. In multivariate analysis, likelihood of no reperfusion therapy was a function of PPCI accessibility (odds ratio [OR] for fibrinolysis versus PPCI centers = 3.1; 95% CI: 2.2–4.4), presence of LBBB (OR = 24.1; 95% CI: 17.8–32.9) and an ECG ambiguous for STEMI (OR = 4.1; 95% CI: 3.3–5.1). When the ECG was ambiguous, likelihood of no reperfusion therapy was highest in hospitals most distant from PPCI centers.

**Conclusions:**

ECG diagnostic ambiguity, LBBB and PPCI accessibility are important predictors of not receiving reperfusion therapy, suggesting opportunities for improving outcomes.

## Introduction

The standard of care for patients presenting with ST-elevation myocardial infarction (STEMI) is immediate reperfusion with primary percutaneous coronary intervention (PPCI) or fibrinolysis [1], [2]. The prognosis of patients with STEMI is better if these interventions can be performed expeditiously (for PPCI, door-to-device within 90 minutes and for fibrinolysis, door-to-needle within 30 minutes). When both options are readily available, PPCI is favored if timely. While many clinical studies have compared choice of treatment and examined timeliness of reperfusion, a more important healthcare gap may be the substantial proportion of patients with STEMI who do not receive any reperfusion therapy at all [3]. Registry data suggest that as many as 1 in 3 patients with STEMI receive neither PPCI nor fibrinolysis and that these patients have a mortality risk several times greater than patients who do receive reperfusion treatment [4]–[7].

Most previous investigations of non-receipt of reperfusion therapy have focused on patient clinical features. Less is known about the role of processes of care, such as a hospital’s predominant reperfusion strategy and access to PPCI. Among centers that receive patients with STEMI, there are those that exclusively treat with on-site PPCI and others at great distances from PPCI facilities that can only treat with fibrinolysis. Other centers without on-site cardiac catheterization laboratories exclusively transfer their patients to nearby sites for PPCI, and a fourth type of center uses a varying mix of transfer for PPCI and on-site fibrinolysis. Besides patient clinical factors, these distinct clinical care environments may influence who receives and who does not receive reperfusion treatment. Less is also known about the impact on non-receipt of reperfusion therapy of electrocardiograms (ECGs) that show left bundle branch block (LBBB) or that do not present a clear-cut STEMI diagnosis. Our study objectives were to ascertain the incidence and predictors of non-receipt of reperfusion therapy in different types of hospitals, with particular attention to the presence of ‘difficult-to-interpret’ ECGs.

## Methods

### Ethics Statement

Approval for the study was obtained from the Quebec Commission for Access to Information ethics board, which waived patient consent as neither patient intervention nor contact was involved.

### Study Design

Our field evaluation was an observational cohort study constituted during two 6-month periods – the first from October 1, 2006 to March 31, 2007 and the second from October 1, 2008 to March 31, 2009– and included all acute care hospitals in Quebec, Canada (n = 80 in the first and n = 82 in the second period) that treated at least 30 patients with acute myocardial infarction (AMI) in the previous year. Together, these centers treat more than 95% of all patients with AMI in Quebec (population 7.8 million persons in 2008). The non-consecutive study periods were chosen for periodic auditing purposes and were representative of regular (rather than summer) staffing.

### Data Collection and Patient Identification

**Figure 1:**
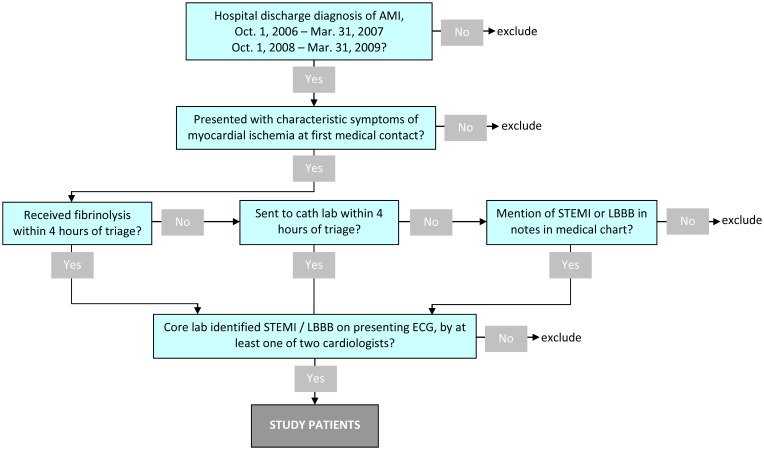
Algorithm to identify study patients. Abbreviations: AMI: acute myocardial infarction. cath lab: cardiac catheterization laboratory. STEMI: ST-segment elevation myocardial infarction. LBBB: left bundle branch block. ECG: electrocardiogram.

The data collection process has been previously detailed in a study that focused on treatment delays and clinical outcomes among STEMI patients who were sent for PPCI or received fibrinolytic therapy in the 2006–7 period [8]. In summary, a certified medical record librarian was designated at each hospital and individually trained for this project. A standardized data collection process was used to abstract all information from medical charts and enter data onto a secure centralized Web site [9]. Using an algorithm (Figure 1), we included all STEMI patients who did and did not receive reperfusion therapy by identifying all patients in the study periods who had a final hospital discharge diagnosis of AMI (ICD-10 codes: I21– I22.9; I24) and presented with characteristic symptoms of acute myocardial ischemia at first medical contact. All patients included were: 1) treated with fibrinolysis within 4 hours of triage, or 2) sent for PPCI within 4 hours of triage, or 3) were not in groups 1 or 2 but had mention of STEMI or LBBB in their medical chart. In addition, all patients had to have a presenting ECG showing either 1) diagnostic ST-segment elevation or 2) LBBB according to at least one of the two cardiologists at the study core laboratory.

### Variables and Outcomes

For the purposes of this study, patients with retrospectively validated ECG eligibility for reperfusion treatment who neither received fibrinolysis nor were sent to a PPCI laboratory within 4 hours of triage were considered to have not received reperfusion therapy. We set this particular time window since pilot tests showed it was highly unlikely for patients with a first ECG showing STEMI to be treated with reperfusion therapy after more than a 4-hour delay. Clinical factors examined were age, sex, systolic blood pressure at triage, heart rate at first in-hospital ECG, and symptom duration to triage. For the ECG interpretation variable, patients without LBBB were categorized as 1) a ‘definite’ STEMI diagnosis when both study cardiologists agreed that STEMI was present: yes/yes; or 2) an ‘ambiguous’ STEMI diagnosis, when only one cardiologist felt STEMI was present, while the other scored not STEMI or was uncertain: yes/no or yes/uncertain). The first ECG was also classified according to presence or absence of anterior STEMI for patients without LBBB.

The acute care hospitals were grouped into 4 distinct mutually exclusive categories based on reperfusion strategy [10]: 1) PPCI centers (i.e., the predominant [≥95%] reperfusion treatment was on-site PPCI); 2) routine transfer PPCI centers (i.e., non-PPCI centers that predominantly [≥95%] transferred their STEMI patients for PPCI); 3) mixed (non-PPCI) centers that used both transfer for PPCI and on-site fibrinolysis as reperfusion strategies (with a mean of 64% of STEMI patients being transferred for PPCI and 34% receiving fibrinolysis); and 4) routine fibrinolysis centers (i.e., non-PPCI centers that predominantly [≥95%] treated their STEMI patients with fibrinolysis). There is no pre-hospital fibrinolysis in Quebec. We calculated the median distances of the three non-PPCI types of centers to the nearest PPCI facility as an index of PPCI accessibility. Other process-of-care variables included triage outside of regular work hours (6∶00 p.m. to 7∶59 a.m., 7 days/week), triage on the weekend, and arrival at first emergency room by self-transport (versus ambulance). Volume of STEMI during the 6-month study periods was categorized as low (≤10), medium (11–25) and high (>25) admissions.

Patient comorbidities were ascertained via Quebec’s administrative hospital discharge database whose reliability has been shown [11]. The recorded principal diagnosis and up to 15 secondary diagnoses were examined for the index hospital admission as well as for all other hospital admissions during the 5 years prior to the index. Only diagnoses considered chronic were ascertained from the index admission, to avoid misclassifying possible acute complications as comorbidities. The Charlson comorbidity index was calculated for each patient and categorized as 0, 1, 2, 3, 4, and 5 or more [12].

### Statistical Analysis

The distribution of demographic and clinical factors, ECG characteristics, and hospital variables was compared in patients who received reperfusion therapy versus patients who did not, using Pearson’s chi-square test for categorical variables or the Kruskal-Wallis test for continuous variables. A crude odds ratio (OR) and the 95% Wald confidence interval (CI) for the association between each variable and non-receipt of reperfusion therapy were calculated.

To develop a parsimonious logistic regression model for the prediction of non-receipt of reperfusion therapy, we used a forward selection procedure beginning with a model that included *a priori*, age, systolic blood pressure, heart rate, type of center and study period. The candidate variable (Table 1) with the highest predictive value was then added incrementally, until additional variables failed to improve the model Akaike Information Criterion. In order to ensure that our inferences were equally valid for both periods, we measured the model fit for both study periods using the c-statistic [13]. As a sensitivity analysis, we also ran a multilevel logistic regression with the same fixed effects as the multivariate model above, but with the addition of 82 random-effects intercepts corresponding to each hospital center.

**Table 1:** Univariate Predictors of Non-Receipt of Reperfusion Therapy (N = 3730).

Description of Variables	Reperfusion Therapy (n = 2918)		No Reperfusion Therapy (n = 812)		Univariate OddsRatio (95% CI)	p
	N	%	N	%		
Age (years), median (IQR)	60 (52–70)		77 (63–84)		1.41 (1.36–1.46) *	<0.001
Female sex	725	24.8	360	44.3	2.41 (2.05–2.83)	<0.001
Heart rate >100 beats/min	328	11.2	236	29.1	3.24 (2.67–3.91)	<0.001
Systolic blood pressure ≥100 mm Hg	2572	88.1	729	89.8	1.18 (0.92–1.53)	0.20
Symptom duration >3 h	844	28.9	457	56.3	3.16 (2.70–3.71)	<0.001
Left bundle branch block (LBBB)						
Absent	2820	96.6	445	54.8	Reference	
Present	98	3.4	367	45.2	23.73 (18.66–30.43)	<0.001
ECG Interpretation (no LBBB)						
Definite STEMI	2154	76.4	188	42.2	Reference	
Ambiguous STEMI	666	23.6	257	57.8	4.42 (3.60–5.44)	<0.001
Anterior MI (no LBBB)						
Absent	1911	67.8	299	67.2	Reference	
Present	909	32.2	146	32.8	1.03 (0.83–1.27)	0.78
Charlson comorbidity index, median (IQR)						
Odds ratio per 1 point increment	1 (0–2)		2 (0–4)		1.67 (1.58–1.76)	<0.001
Triage outside of regular work hours	1302	44.6	410	50.5	1.27 (1.08–1.48)	0.003
Triage on weekend	865	29.6	242	29.8	1.01 (0.85–1.19)	0.93
Arrival by self-transport	1043	35.7	274	33.7	0.92 (0.78–1.08)	0.29
Center STEMI Volume						
High (>25 admissions)	2007	68.8	506	62.3	Reference	
Medium (11–25)	665	22.8	211	26.0	1.26 (1.05–1.51)	
Low (10)	246	8.4	95	11.7	1.54 (1.18–1.97)	<0.001
Type of Center						
PPCI center	923	31.6	185	22.8	Reference	
Transfer PPCI center	969	33.2	262	32.3	1.35 (1.10–1.66)	
Mixed center	748	25.6	248	30.5	1.65 (1.34–2.05)	
Fibrinolysis center	278	9.5	117	14.4	2.10 (1.60–2.74)	<0.001

*odds ratio for each 5-year age increment; CI: confidence interval; ECG: electrocardiogram; h: hours; IQR: interquartile range; LBBB: left bundle branch block; MI: myocardial infarction; min: minute; PPCI: primary percutaneous coronary intervention; STEMI: ST-elevation myocardial infarction.

In order to investigate the differences across centers in terms of patient factors associated with non-receipt of reperfusion therapy, we applied the same multivariate model developed for the overall cohort (minus the variable for type of center) to patients presenting to each of the 4 hospital types. We used meta-analytic techniques in order to consider the differences in effects across the 4 center types; specifically, we used Cochran’s Q statistic to assess the statistical significance of heterogeneity and Higgins’ I^2^ statistic to assess the magnitude of heterogeneity [14]. We classified heterogeneity as low (I^2^≤33%), medium (33%<I^2^≤66%) or high (I^2^>66%). We report two-sided p-values, and considered the threshold for statistical significance to be 5%. Statistical analyses were performed using R software version 2.15 [15].

## Results

We examined the medical charts of 14,781 patients who presented to an emergency room with characteristic symptoms in the two study periods and who had a final discharge diagnosis of AMI. On the basis of the algorithm and the core-laboratory analysis of the first presenting ECG, 3730 (25.2%) of these patients were classified as having STEMI/LBBB, of whom 1897 presented in the first period and 1833 presented in the second. Of the 3730 patients, 465 patients (12.5%) had LBBB, 2342 patients (62.8%) had a definite STEMI ECG and 923 patients (24.7%) had an ECG considered ambiguous for STEMI.

Of all 3730 patients, the proportions presenting to the 4 types of hospitals were 29.7% to PPCI centers, 33.0% to transfer PPCI centers, 26.7% to mixed centers and 10.6% to fibrinolysis centers. Median distances to the nearest PPCI center were 20 km (inter-quartile range, IQR: 7–39) for transfer PPCI centers, 77 km (IQR: 23–126) for mixed centers, and 307 km (IQR: 131–488) for fibrinolysis centers (p<0.001). The proportions presenting with an ambiguous STEMI ECG (and no LBBB) were 29.1% in PPCI centers, 29.8% in transfer PPCI centers, 28.1% in mixed centers and 21.9% in fibrinolysis centers (p = 0.04). The proportion of STEMI/LBBB patients who neither received fibrinolysis nor were sent for PPCI within 4 hours of emergency room triage was 21.8% overall, decreasing from 23.5% to 20.0% across the study periods (p = 0.009).

### Univariate Predictors of Non-Receipt of Reperfusion Therapy (Table 1)

Patients with STEMI/LBBB who did not receive reperfusion therapy were significantly older, more likely to be female, more often had a heart rate >100/min, and had a longer duration of symptoms before presentation compared with those who received reperfusion therapy. Each 5-year increase in age was associated with greater likelihood of no reperfusion therapy (OR = 1.41; 95% CI: 1.36–1.46). Cardiac and non-cardiac comorbidities were more frequent in patients who did not receive reperfusion treatment (not shown in Table); accordingly, the median Charlson comorbidity score was higher for those without reperfusion therapy (p<0.001).

LBBB was present in 45.2% (367/812) of patients not receiving reperfusion therapy, compared with 3.4% (98/2918) in patients who received reperfusion treatment (OR = 23.76; 95% CI: 18.66–30.43). Among patients without LBBB but with an ECG that was ambiguous for STEMI, the odds of not receiving reperfusion therapy was 4.42 (95% CI: 3.60–5.44) times higher than in patients with an ECG that was a definite STEMI diagnosis.

The likelihood of non-receipt of reperfusion treatment varied significantly by accessibility to PPCI. Of 1108 STEMI/LBBB patients presenting directly to a PPCI center, 185 patients (16.7%) did not receive reperfusion therapy compared with 21.3% (262/1231) for transfer PPCI centers, 24.9% (248/996) for mixed centers, and 29.6% (117/395) for fibrinolysis centers (p<0.001). Other factors found to be associated with no reperfusion were lower center volume and triage outside of regular work hours.

### Multivariate Predictors of Non-Receipt of Reperfusion Therapy (Table 2)

In multivariate analysis, less accessibility to PPCI, LBBB and an ECG that was ambiguous for STEMI were associated with non-receipt of reperfusion therapy. The odds of not receiving reperfusion therapy were very similar for PPCI centers and centers that exclusively transferred their STEMI patients for PPCI. However, patients with STEMI/LBBB who presented to centers that treated exclusively with fibrinolysis had 3-fold higher odds of not receiving reperfusion therapy. Patients presenting to mixed centers that were geographically closer to a PPCI facility than the exclusively fibrinolytic centers but further away than sites systematically transferring their patients for PPCI, had intermediate, 1.5-fold odds of not receiving therapy. The odds of not receiving reperfusion therapy were especially pronounced for patients who had an ECG ambiguous for STEMI (excluding LBBB; OR = 4.07; 95% CI: 3.25–5.12) and patients who presented with LBBB (OR = 24.10; 95% CI: 17.81–32.89).

Other factors in the multivariate model associated with no reperfusion treatment were: older age; female sex; higher systolic blood pressure (≥100 mm Hg); higher heart rate (>100 beats/min); longer symptom duration; higher Charlson comorbidity index; self-transport to emergency room; and earlier observation period. Triage outside of regular work hours was borderline significant. Elevated c-statistics of 0.87 for the first study period and 0.89 for the second show that the model was consistently able to discriminate patients likely to receive reperfusion therapy from patients unlikely to receive treatment. In the sensitivity analysis using a multilevel approach, the odds ratios and confidence intervals in Table 2 remained substantively unchanged.

**Table 2:** Multivariate Predictors of Non-Receipt of Reperfusion Therapy (N = 3730).

Variable	Odds Ratio (95% CI)
Age (per 5-year increment)	1.21 (1.16–1.27)
Female sex (versus male)	1.71 (1.37–2.14)
Systolic blood pressure ≥100 mm Hg (versus <100 mm Hg)	1.44 (1.03–2.03)
Heart rate >100 beats/min (versus ≤100 beats/min	1.36 (1.04–1.76)
Symptom duration >3 h (versus ≤3 h)	2.13 (1.73–2.62)
**ECG Interpretation**	
Definite STEMI (no LBBB)	Reference
Ambiguous STEMI (no LBBB)	4.07 (3.25–5.12)
Left bundle branch block (versus absence)	24.1 (17.81–32.89)
**Charlson Comorbidity Index**	
Per 1 point increment	1.30 (1.21–1.40)
Triage outside of regular work hours (versus during)	1.20 (0.98–1.47)
Arrival by self-transport (versus ambulance)	1.42 (1.13–1.78)
**Type of Center**	
PPCI center	Reference
Transfer PPCI center	0.98 (0.75–1.29)
Mixed center	1.53 (1.16–2.02)
Fibrinolysis center	3.10 (2.20–4.37)
Period 2008–9 (versus 2006–7)	0.67 (0.54–0.82)

Abbreviations as in Table 1.

### Predictors of Non-Receipt of Reperfusion Therapy by Type of Center (Table 3)

When the same predictive model of non-receipt of reperfusion therapy was applied by center type, the statistical discrimination was excellent (c-statistic ≥0.87). The models generally confirmed that the types of factors impacting non-receipt of reperfusion therapy were similar in all types of centers, with some possible heterogeneity in the estimates of association for female sex, ambiguous ECG and cohort period.

**Table 3:** Multivariate Predictors of Non-Receipt of Reperfusion Therapy, by Type of Center (N = 3730).

	Odds Ratio (95% CI)				Heterogeneity	
Variable	PPCI Centers	Transfer PPCI Centers	Mixed Centers	Fibrinolysis Centers	I^2^ (95% CI)	p
Age (per 5-year increment)	1.31 (1.20–1.43)	1.20 (1.11–1.29)	1.16 (1.07–1.26)	1.27 (1.13–1.43)	36 (0–78)	0.32
Female sex	1.62 (1.01–2.59)	1.93 (1.32–2.83)	2.32 (1.52–3.55)	0.78 (0.39–1.52)	60 (0–87)	0.11
Systolic blood pressure ≥100 mm Hg	1.98 (1.00–4.08)	2.09 (1.18–3.82)	0.95 (0.52–1.82)	1.02 (0.38–3.07)	31 (0–75)	0.36
Heart rate >100 beats/min	1.03 (0.60–1.75)	1.40 (0.90–2.13)	2.09 (1.25–3.47)	0.73 (0.28–1.80)	45 (0–82)	0.24
Symptom duration >3 h	1.55 (1.01–2.36)	1.98 (1.38–2.86)	2.64 (1.77–3.95)	3.34 (1.85–6.12)	47 (0–82)	0.23
**ECG Interpretation**						
Ambiguous STEMI (no LBBB)	7.66 (4.77–12.56)	3.52 (2.34–5.35)	2.76 (1.81–4.22)	5.84 (3.17–10.90)	74 (27–91)	0.02
Left bundle branch block	34.82 (18.91–66.42)	22.58 (13.82–37.65)	27.14 (14.46–53.65)	24.32 (8.61–79.97)	0 (0–60)	0.89
**Charlson Comorbidity Index**						
Per 1 point increment	1.34 (1.16–1.53)	1.3 (1.15–1.48)	1.32 (1.15–1.52)	1.36 (1.10–1.68)	0 (0–0)	1.00
Triage outside of regular work hours	1.69 (1.11–2.57)	1.14 (0.80–1.64)	0.96 (0.64–1.42)	1 (0.56–1.75)	29 (0–74)	0.38
Arrival by self-transport	1.25 (0.78–2.00)	1.45 (0.98–2.16)	1.44 (0.93–2.23)	1.44 (0.79–2.64)	0 (0–0)	0.99
Period 2008–9 (versus 2006–7)	0.47 (0.30–0.72)	0.56 (0.38–0.82)	1.1 (0.74–1.65)	0.61 (0.34–1.08)	69 (9–89)	0.05

Abbreviations as in Table 1.

### Association of ECG Interpretation and Non-Receipt of Reperfusion Therapy by Type of Center (Table 4)

For patients with a definite STEMI ECG (and no LBBB), a statistically significant gradient for receipt of reperfusion therapy was observed, from over 95% of patients in PPCI centers to 85.5% in fibrinolysis centers, consistent with the degree of access to PPCI. When the ECG was ambiguous for STEMI (but no LBBB), a quarter of patients in PPCI centers did not receive reperfusion therapy compared to over half of patients presenting to exclusively fibrinolytic centers (p<0.001). Most patients with LBBB in all types of centers did not receive reperfusion therapy. However, a gradient based on accessibility to PPCI again appeared to be present: twice as many patients with LBBB in on-site PPCI and transfer PPCI centers (26.2%) received reperfusion compared with LBBB patients presenting to fibrinolysis and mixed centers (12.6%; p = 0.09).

**Table 4:** Proportion of Patients With Receipt of Reperfusion Therapy (RT) and With Non-Receipt of Reperfusion Therapy (NRT) According to ECG Interpretation and Center Type (N = 3730).

ECG Interpretation	PPCI Centers (n = 1108)		Transfer PPCI Centers (n = 1231)		Mixed Centers (n = 996)		Fibrinolysis Centers (n = 395)		p
	RT (%)	NRT (%)	RT (%)	NRT (%)	RT (%)	NRT (%)	RT (%)	NRT (%)	
Definite STEMI (no LBBB)	678 (95.2)	34 (4.8)	683(93.1)	51 (6.9)	558 (89.9)	63 (10.1)	235 (85.5)	40 (14.5)	<0.001
Ambiguous STEMI (no LBBB)	217 (74.3)	75 (25.7)	238 (76.5)	73 (23.5)	174 (71.6)	69 (28.4)	37 (48.1)	40 (51.9)	<0.001
LBBB	28 (26.9)	76 (73.1)	48 (25.8)	138 (74.2)	16 (12.1)	116 (87.9)	6 (14.0)	37 (86.0)	0.006

Abbreviations as in Table 1.

Denominators for percentages in the Table are the number of patients presenting to each type of center with the ECG interpretation in question.

## Discussion

### Summary of Findings

This comprehensive, system-wide field evaluation of STEMI care allowed us to ascertain the clinical, ECG, and process-of-care characteristics that are associated with the non-receipt of reperfusion therapy. The main study findings are: (1) a substantial proportion of patients with STEMI/LBBB do not receive reperfusion therapy; (2) the likelihood of not receiving reperfusion therapy was a function of accessibility to PPCI, being least in PPCI centers and greatest in fibrinolysis centers; (3) uncertainty of STEMI on presenting ECGs (as suggested by expert ECG review) and presence of LBBB largely predict non-receipt of reperfusion therapy; and (4) the likelihood of not receiving reperfusion therapy in the presence of LBBB or an ECG that was ambiguous for STEMI was higher in centers far removed geographically from PPCI facilities.

### Study Strengths

Previous investigations, based on STEMI registries, have focused primarily on clinical characteristics of patients who have not received reperfusion therapy in PPCI centers [6], [7], [16], [17]. However, many registries do not include all hospitals within a system of care nor necessarily recruit all STEMI/LBBB patients, raising questions of representativeness and generalizability of their findings [16]–[18]. Even when patients who are transferred in for PPCI from non-tertiary centers are included [6], [7], [19], patients who are not transferred are not identified. Moreover, registries tend not to have centralized ECG interpretation to standardize identification of all STEMI patients, and have not generally considered whether ECG characteristics – such as the degree of certainty of STEMI and the presence of LBBB – are associated with the likelihood of not receiving reperfusion treatment. Finally, previous studies have not focused on non-receipt of reperfusion therapy from the perspectives of hospital reperfusion strategy and PPCI accessibility.

The major strength of our study is its systematic and comprehensive recruitment of patients within a complete system of cardiac care that includes all centers regardless of their predominant reperfusion treatment and includes both patients who did and did not receive reperfusion, and patients who were not transferred. Other relatively novel strengths are the centralized ECG validation, the inclusion of patients with LBBB, the inclusion of transfer-in and transfer-out patients, and the inclusion of process-of-care variables in the analysis, such as time period of triage, means of transport to hospital, and hospital reperfusion practice as well as hospital STEMI volume. An important challenge in examining the problem of non-receipt of reperfusion treatment in patients with STEMI is how to define the patients eligible for reperfusion in terms of ECG characteristics. Without systematic centralized ECG reading, it is uncertain in a given study if all eligible STEMI patients have been identified; therefore, it cannot be certain that the patients who have not received reperfusion treatment have been correctly coded. Our study was designed to address this difficulty by requiring at least one of the two cardiologists at the core laboratory to confirm that STEMI was present. This also allowed us to dichotomize ECGs with STEMI into either definite or ambiguous categories.

### The Interplay of Clinical, ECG and Hospital Factors

Our study shows that patient and hospital characteristics both independently contribute to the likelihood of non-receipt of reperfusion treatment. Patient characteristics independently associated with non-receipt of reperfusion therapy included older age, female sex, high heart rate, higher Charlson index, and longer symptom duration. Two important and independent ECG predictors of not receiving reperfusion were LBBB and, in patients without LBBB, an ECG that appeared to be ambiguous for STEMI, as defined by a lack of consensus by 2 reviewing cardiologists. And in the presence of these ECG features in patients presenting with symptoms of AMI, the rate of reperfusion therapy was about twice as high in patients presenting to centers with ready access to PPCI either because it was on site or because transfer for PPCI was a feasible option. This is consistent with guidelines recommending prompt coronary angiography when the diagnosis of STEMI is uncertain [20].

LBBB was present in 12.5% of the study cohort. Although it has been shown that the presence of LBBB in patients presenting with acute symptoms is a relatively insensitive marker of AMI [21], this finding does not strictly apply to our patients since the entry criteria in our field evaluation required a hospital discharge summary AMI diagnosis. Patients with LBBB comprised nearly half of our patients with AMI who did not receive reperfusion treatment. While older guidelines consistently recommended that patients with new or presumably new LBBB and characteristic symptoms receive reperfusion treatment just like patients with classic STEMI [1], [2], [20], more recent guidelines are conflicting [22], [23]. Insofar as only 98 of 465 AMI patients with LBBB (21.1%) in our field evaluation actually received reperfusion treatment, from the perspective of the guidelines recommending that new or presumably new LBBB be treated as a STEMI equivalent, these findings appear to reveal an important treatment gap. On the other hand, despite the strength of the recommendation (Class IA) [20], [23], the benefit of reperfusion therapy in patients with new or presumably new LBBB does not seem to have ever been prospectively tested. The recommendation is based on sub-group meta-analytic data from the Fibrinolytic Therapy Trialists’ Collaborative Group [24], showing that patients with bundle branch block derived even greater absolute benefit from fibrinolysis than patients with classic ST-elevation. However, it is not known whether and in what proportions these patients with unspecified bundle branch block actually had LBBB versus right bundle branch block, new or presumed new or old bundle branch block, or ST-elevation or any more suggestive indications of STEMI on an ECG showing LBBB [25]. Therefore, the manifest reluctance of clinicians to treat patients with suspected myocardial infarction and LBBB with reperfusion therapy might be more due to a knowledge gap in the evidence base than to a treatment gap, *per se*. Indeed, these considerations, added to concern over both fibrinolytic risk and false system activation for PPCI in many patients presenting with acute symptoms and LBBB (who turn out not to have an acutely occluded coronary artery), have resulted in the 2013 AHA/ACC STEMI guidelines questioning the notion of the LBBB-STEMI equivalent [22]. Because patients who present with myocardial infarction and LBBB are at extremely high risk [26] and only infrequently trigger a ‘reperfusion reflex’ on the part of clinicians, the question is raised whether the benefit of reperfusion treatment should not be prospectively tested perhaps using novel triage algorithms [21], [27] in this relatively important and high-risk subset.

Apart from patients with LBBB, a quarter of study patients had a presenting ECG judged ambiguous for STEMI by the study cardiologists, and about one third of these patients did not receive any reperfusion treatment. These results underscore that an important proportion of patients with STEMI or presumptive STEMI have presenting ECGs where the diagnosis is not clear-cut, even for cardiologists [28]. Such findings suggest that an underappreciated reason for not receiving reperfusion therapy may be the understandable difficulty that physicians in emergency rooms face in the interpretation of these challenging ECGs, a difficulty that must be considerably greater than that encountered by cardiologists. Our findings point to the need for support systems enabling emergency physicians to have access to immediate and expert ECG opinion, and the importance of continuing education initiatives focused on challenging ECGs. While the helpfulness of expert advice may be questioned if the experts themselves do not always agree (as in this study), it should be noted that the study cardiologists based their retrospective evaluations of these ECGs in isolation from the patients’ acute clinical presentations. It is likely that immediate consultation in the presence of difficult acute ECGs – in which contextual clinical information is also shared – would be of considerable assistance to emergency physicians, and would decrease the rate of non-receipt of reperfusion therapy.

At the hospital level, the rate of non-receipt of reperfusion was higher in fibrinolysis centers. In addition to more difficult access to expert ECG interpretation, this finding is likely due to the real and perceived risks of bleeding, especially intra-cerebral hemorrhage, associated with fibrinolysis and far less so with PPCI. It would thus be expected that the threshold for initiating fibrinolysis would be higher than the decision to send a STEMI patient for PPCI. This latter option was precluded in these distant, low-volume centers.

### Cohort Period

In the second observation period, 2 years after the first, the proportion of patients who were not sent for PPCI and who did not receive fibrinolytic therapy decreased significantly by 15%. Indeed, the later period was associated in multivariate analysis with a third less chance of not receiving reperfusion treatment. This improvement may be attributed to greater physician awareness of the importance of not withholding reperfusion treatment from eligible patients. Publications highlighting this treatment gap and its adverse implications [3]–[5], [7], the direct dissemination of findings from our first evaluation to all health regions and hospitals, our policy guidance document [29], as well as other research [30] and quality improvement initiatives in the province, are likely to have contributed to this salutary change. Interestingly, this improvement occurred in all types of centers except mixed perfusion treatment centers (Table 3). This might point to the difficulty of effecting change in centers that do not have a consistent reperfusion strategy.

### Study Limitations

We were unable to comprehensively measure other clinical or process-of-care variables that may be related to the decision not to provide reperfusion therapy. These include real and perceived bleeding risks (especially for patients whose only reperfusion option, based on hospital type, is fibrinolysis), patient or family refusal, clinical judgment in fragile patients who often have considerable comorbidity that aggressive treatment would be futile or inappropriate, hospital STEMI protocols and the number of cardiologists, and the ratio of specialists to general practitioners per center. However, we likely did at least indirectly capture some of these reasons for non-receipt of reperfusion with several of our independent predictors, such as older age, higher Charlson index, and type of center. We did not integrate cardiac enzyme and biomarker data or coronary angiographic data in our analyses. Nevertheless, biomarker information is integral to physician and, by extension, ICD-10 AMI diagnosis [31] that was the starting point for our identification of STEMI patients. We did not characterize certain population-level factors like income, race and population density; however, their impact may be less important within a universal public health system. We only considered the first presenting ECG although we recognize that, in some clinical presentations, appropriate diagnosis and treatment require serial ECG analysis. Finally, with regard to LBBB, first, we could not discriminate between new and old LBBB, although we believe this distinction is of uncertain significance and, in practical terms, is generally problematic in the acute clinical setting. Second, while all LBBB patients in this field evaluation had a final diagnosis of AMI, we cannot exclude that at least some of them may not have had a STEMI (or non-STEMI) equivalent but rather heart failure with a rise in necrosis biomarkers. This limitation only underlines the challenge facing clinicians when patients with LBBB present with symptoms compatible with myocardial ischemia. A diagnostic coronary angiogram may be the only way to appropriately triage such high-risk patients and it is pertinent that in our study twice as many patients with LBBB received reperfusion treatment if timely accessibility to coronary catheterization was possible.

### Conclusions

In conclusion, by integrating clinical, ECG, and process-of-care data within a complete system of STEMI care, our novel field evaluation has furnished important and novel insights into the conundrum of patients with suspected STEMI who do not receive reperfusion treatment. An implication of this research is that the development of programs and training for emergency room physicians in order to correctly deal with ambiguous or difficult-to-interpret ECGs and LBBB is necessary, and should be tailored to a given hospital’s proximity and access to coronary angiography centers. Awareness and greater understanding of these issues will likely improve patient care and reduce mortality in this important and very high-risk group.
